# Case Report: Omicron BA.2 Subvariant of SARS-CoV-2 Outcompetes BA.1 in Two Co-infection Cases

**DOI:** 10.3389/fgene.2022.892682

**Published:** 2022-04-12

**Authors:** Marija Gjorgjievska, Sanja Mehandziska, Aleksandra Stajkovska, Slavica Pecioska-Dokuzovska, Anica Dimovska, Idriz Durmish, Sara Ismail, Teodora Pavlovska, Antonija Stojchevska, Haris Amedi, Jasna Andonova, Marija Nikolovska, Sara Velickovikj, Zan Mitrev, Ivan Kungulovski, Goran Kungulovski

**Affiliations:** ^1^ Zan Mitrev Clinic, Skopje, North Macedonia; ^2^ Bio Engineering LLC, Skopje, North Macedonia

**Keywords:** COVID-19, co-infection, omicron, next-generation sequencing, SARS- CoV2

## Abstract

Trends from around the world suggest that the omicron BA.2 subvariant is increasing in proportion to the original BA.1 subvariant. Here we report two cases of co-infection with omicron BA.1 and omicron BA.2 in co-exposed individuals. In both individuals, genome sequencing and/or S-gene specific PCR identified omicron BA.1 at early time-points, which was replaced by omicron BA.2 at later time-points of the infection. The timeline of our data supports the proposition that BA.2 outcompetes BA.1 in a real-life scenario, and in time becomes the dominant variant in the upper respiratory tract of the host.

## Introduction

Coronavirus disease 19 (COVID-19) is caused by the severe acute respiratory syndrome coronavirus 2 (SARS-CoV-2), first identified in Wuhan, China (Zhu et al., 2020). Throughout the pandemic, the SARS-CoV-2 virus has been continuously evolving leading to the emergence of new variants. The ones that posed an increased risk to global public health due to increased transmissibility, increased virulence, or immune evasion have been designated as variants of interest (VOIs) or variants of concern (VOCs) by the World Health Organization (https://www.who.int/en/activities/tracking-SARS-CoV-2-variants/) (Otto et al., 2021). The VOCs Alpha, Beta, Gamma, and Delta were first detected in the second half of 2020, with Delta becoming the dominant variant for most of the second half of 2021. By the end of 2021, the omicron variant (BA.1) began overtaking the delta variant as the dominant strain, and by early 2022 it has become the dominant strain in Europe and USA due to its striking antibody evasion properties (Liu et al., 2022). Several countries, including Denmark, have observed two omicron subvariants (BA.1 and BA.2). Evidence from Denmark suggests that omicron BA.2 leads to 2-3 times increased susceptibility to infection compared to BA.1, and has rapidly replaced BA.1 as the dominant subvariant (Lyngse et al., 2022). In line with this evidence, we report two cases of co-infection with omicron BA.1 and omicron BA.2 in co-exposed individuals. In both individuals, genome sequencing and/or S-gene specific PCR identified omicron BA.1 at early time-points, which was replaced by omicron BA.2 at later time-points of the infection.

## Case Presentation

**FIGURE 1:**
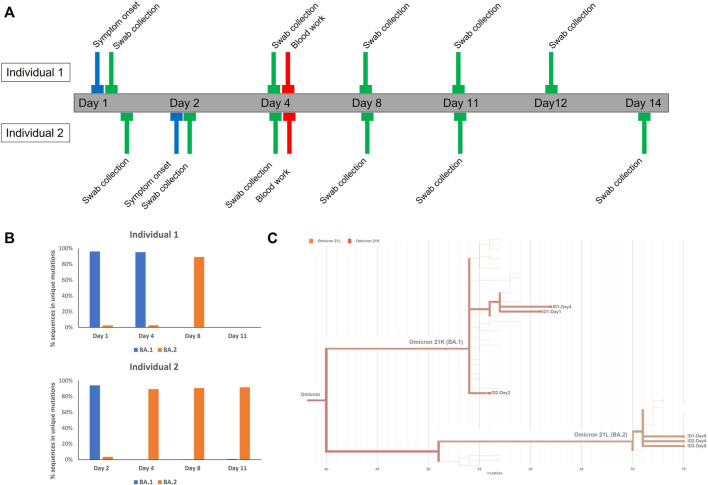
**(A)** Roadmap of sample collection per tested individuals **(B)** Quantification of sequences mapping to unique regions in Omicron BA.1 and omicron BA.2 per day. More information can be found in Supplementary Datasheet S2. **(C)** Phylogenetic tree of genome assemblies.

Two individuals with COVID-19 symptoms were confirmed to be positive by PCR and then analyzed by genome sequencing at different time points at our institution (Figure 1A). First, a 25-year-old female (individual 1), which experienced throat scratching and low-grade fever on day 1, underwent SARS-CoV-2 PCR confirmatory testing after getting a positive lateral flow test. The assay resulted in a positive PCR test with S-gene target failure (SGTF), followed by another SGTF PCR test on day 4 (Table 1; Supplementary Figure S1). Interestingly, the follow-up PCR test on day 8 exhibited S-gene amplification (non-SGTF) (Table 1, Supplementary Figure S1). Consistent with the PCR results, SARS-CoV-2 genome sequencing confirmed the presence of omicron BA.1 in the samples collected on day 1 and day 4, and omicron BA.2 on day 8 (Figures 1B,C; Supplementary Figure S2). Unfortunately, samples were not collected on days 9 and 10, whilst PCR samples on days 11 and Day 12 were negative.

**TABLE 1:** Timeline of symptom emergence, PCR results, SGTF status and genome sequencing.

		Day 1	Day 2	Day 4	Day 8	Day 11	Day 12	Day 14
Individual 1	Ct (ORF1ab)	25.5	/	24.5	23	NEG	NEG	/
	SGTF (PCR)	YES	/	YES	NO	NEG	NEG	/
	NGS	Omicron BA.1	/	Omicron BA.1	Omicron BA.2	/	/	/
	Symptoms	YES	YES	NO	NO	NO	NO	NO
Individual 2	Ct (ORF1ab)	34	27.5	19	21	24.5	/	29.5
	SGTF (PCR)	NO	NO	NO	NO	NO	/	NO
	NGS	/	Omicron BA.1	Omicron BA.2	Omicron BA.2	/	/	/
	Symptoms	NO	YES	NO	NO	NO	NO	NO

Her partner, a 35-year-old male (individual 2) tested positive at day 1 for trace amounts of SARS-CoV-2, yielding a non-SGTF PCR, which was confirmed by follow-up non-SGTF PCRs at day 2, day 4, day 8, day 11, and Day 14 (Table 1; Supplementary Figure S1). He experienced symptom onset at day 2 manifested as throat discomfort early in the day and fever in the evening. Surprisingly, genome sequencing at day 2 and genome assembly analysis indicated infection with the omicron BA.1 subvariant, in contrast to the PCR result. More focused analysis of selected sequences in regions specific for BA.1 and BA.2 revealed the presence of sequences specific for both genomes, with the majority of sequences belonging to BA.1 (Figure 1B; Supplementary Figure S2). Due to gaps in genome sequencing and incomplete genome assembly (e.g the 69-70 deletion region was not covered), we were unable to carry out more precise comparative analyses, which might further explain the discrepancy between the sequencing and PCR results; only 5/15 BA.1, and 12/23 BA.2 mutations were covered with >10 sequences in this sample (Supplementary Material S2). Further sequencing at day 4 and day 8 unequivocally identified the BA.2 subvariant with none of the BA.1 specific sequences present in our samples (Figures 1B,C; Supplementary Figure S2).

Both individuals were fully vaccinated with two doses of the BNT162b2, COVID-19 mRNA vaccine, 9 and 11 months prior to the infection, respectively. The most likely route of omicron BA.1 infection for individual 1 was a beauty salon; individual 2 was most likely exposed to omicron BA.2 in a crowded restaurant. Both individuals underwent home isolation together and experienced mild symptoms. Blood work results at day 4 were within reference ranges.

## Materials and Methods

### RNA Extraction and RT-PCR

For the detection of viral RNA by RT-PCR and sequencing, nasal and oropharyngeal swabs were collected for each time point. Both swabs were combined and immersed in saline solution and processed immediately. RNA was extracted with the abGenix (AITbiotech, Singapore) automatic DNA/RNA extractor, and PCR was carried out on QuantStudio™ 5 (Thermo Fisher Scientific, MA, USA) thermal cycler using the TaqPath protocol (ThermoFisher Scientific, MA, USA), according to the manufacturer’s recommendations. Positive and negative controls were routinely included in each run.

### Viral Whole-Genome Sequencing

Reverse transcription using SuperScript IV Reverse Transcriptase (Invitrogen, Thermo Fisher Scientific, MA, USA) and SARS-CoV-2 genome amplification using the ARTIC panel of primers (Integrated DNA Technologies, IA, USA) was performed as described in the Nanopore protocol “*PCR tiling of COVID-19 virus” (Version: PTC_9096_v109_revD_06February 2020)*. The protocol was modified with rapid barcoding instead of native barcoding using the Rapid Barcoding Sequencing Kit, SQK-RBK004 (Oxford Nanopore Technologies, UK). The samples were sequenced on Flow Cell R9.4.1 using the MinION device.

Demultiplexing of the samples was carried out with the FASTQ Barcoding tool on the EPI2ME platform (Oxford Nanopore Technologies, UK). Multiple FASTQ files were concatenated into one, and genome assembly was conducted with the *medaka consensus pipeline* for creating a consensus sequence using Galaxy platform (Afgan et al., 2018). Consensus sequences were used to generate a phylogenetic tree in Nextstrain (https://clades.nextstrain.org/). Genome coverage was between 23x and 121x (Supplementary Figure S3) with two-thirds of bases covered with more than >20 sequences. Finally, editing and gap-filling were done in BioEdit. The coverage of regions specific for omicron BA.1 and omicron BA.2 (Supplementary Datasheet S2) was evaluated directly from the BAM files in the Integrative Genomic Viewer, Broad Institute (Robinson et al., 2017).

## Discussion and Concluding Remarks

In this report, we illustrate two interesting cases of co-infection with omicron BA.1 and omicron BA.2 in co-exposed individuals in the same household. In both individuals, genome sequencing and/or S-gene specific PCR identified omicron BA.1 at early time-points, which was replaced by omicron BA.2 at later time-points of the infection.

The timeline of our sample collection and symptom onset supports the proposition that individual 1 initially got infected with omicron BA.1 and exposed individual 2. During the incubation phase or early phase of infection individual 2 most likely got exposed to omicron BA.2 independently, which quickly outcompeted omicron BA.1 in his upper respiratory tract. During home isolation with individual 2, individual 1 who was already infected with omicron BA.1 got exposed to omicron BA.2, which seems to have outcompeted omicron BA.1 in the following days. These observations suggest that omicron BA.2 has biological properties allowing it to outcompete omicron BA.1 in the host, at least in the immunological and genetic context of these two individuals. Similar cases of co-infection with two different SARS-CoV-2 variants could serve as an evolutionary substrate for viral recombination events, and the emergence of new variants.

In addition, our study has several limitations. The most obvious one is the small sample size of two non-related individuals; meaning that our observations are not readily translatable for other households or larger populations. Second, we were unable to provide complete ungapped genome assembly and full genome coverage of the sample collected on day 2 from individual 2. Unfortunately, this prevented us to conduct more detailed genomic analyses to evaluate the complete distribution and representation of the omicron subvariants in that sample.

All in all, our observations although limited in nature are consistent with the epidemiological situation in several other countries, where omicron BA.2 has replaced or has been replacing omicron BA.1. These studies along with our observations suggest that omicron BA.2 has biological features leading to host-specific growth advantage.

## Data Availability

FASTQ files are available at 10.5281/zenodo.6325649. The genome sequences were deposited in the GISAID database under accession IDs EPI_ISL_11166201, EPI_ISL_11166327, EPI_ISL_11166339, EPI_ISL_11166341, EPI_ISL_11166342, EPI_ISL_11166343.
