# Supplementary material for: Case Report: Omicron BA.2 Subvariant of SARS-CoV-2 Outcompetes BA.1 in Two Co-infection Cases
Source: Front Genet. 2022 Apr 12;13:892682. doi: 10.3389/fgene.2022.892682 (PMC9041751; doi:10.3389/fgene.2022.892682)
Supplement: Supplementary file 1 [file Table1.DOCX]

**Table 1**. Timeline of symptom emergence, PCR results, SGTF status and genome sequencing.

|  |  | **Day 1** | **Day 2** | **Day 4** | **Day 8** | **Day 11** | **Day 12** | **Day 14** |
| --- | --- | --- | --- | --- | --- | --- | --- | --- |
| **Individual 1** | Ct (ORF1ab) | 25.5 | / | 24.5 | 23 | NEG | NEG | / |
|  | SGTF (PCR) | YES | / | YES | NO | NEG | NEG | / |
|  | NGS | Omicron BA.1 | / | Omicron BA.1 | Omicron BA.2 | / | / | / |
|  | Symptoms | YES | YES | NO | NO | NO | NO | NO |
| **Individual 2** | Ct (ORF1ab) | 34 | 27.5 | 19 | 21 | 24.5 | / | 29.5 |
|  | SGTF (PCR) | NO | NO | NO | NO | NO | / | NO |
|  | NGS | / | Omicron BA.1 | Omicron BA.2 | Omicron BA.2 | / | / | / |
|  | Symptoms | NO | YES | NO | NO | NO | NO | NO |
