# Supplementary material for: Case Report: Omicron BA.2 Subvariant of SARS-CoV-2 Outcompetes BA.1 in Two Co-infection Cases
Source: Front Genet. 2022 Apr 12;13:892682. doi: 10.3389/fgene.2022.892682 (PMC9041751; doi:10.3389/fgene.2022.892682)
Supplement: Supplementary file 2 [file DataSheet1.PDF]

Omicron BA.2 subvariant of SARS-COV-2 outcompetes BA.1 in two co-infection cases

Marija Gjorgjievska[1], Sanja Mehandziska[1], Aleksandra Stajkovska[2], Slavica Pecioska-Dokuzovska[1], Anica Dimovska[1], Idriz Durmish[1], Sara Ismail[1], Teodora Pavlovska[1], Antonija Stojcevska[1], Haris Amedi[1], Jasna Andonova[1], Marija Nikolovska[1], Sara Velichkovikj[1], Zan Mitrev[1], Ivan Kungulovski[2], Goran Kungulovski[2*]

[1]Zan Mitrev Clinic, Skopje, Republic of Macedonia; [2]Bio Engineering LLC, Skopje, Republic of Macedonia

[2]Bio Engineering LLC, Skopje, North Macedonia

**Corresponding author:*

Dr. Goran Kungulovski, Bio Engineering LLC, Laboratory of Genetics and Personalized Medicine, Zan Mitrev Clinic

Ivan Agovski 7-1, 1000, Skopje, Republic of Macedonia

Phone: +389 70 234280

goran@bioengineering.mk

goran.kungulovski@zmc.mk

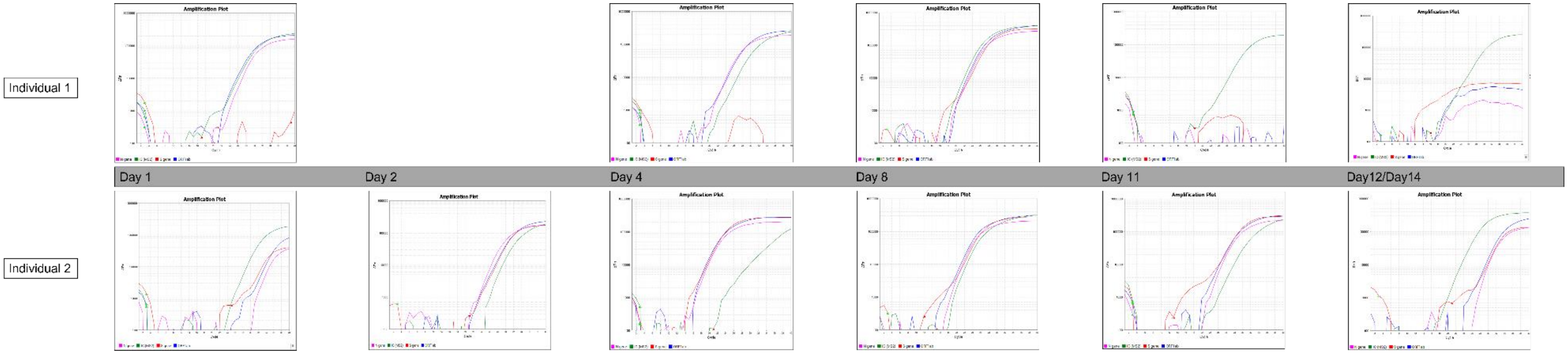

Supplementary Figure 1. Roadmap of PCR experiments in individual 1 and individual 2. Signals of *N-gene* are depicted in purple, *S-gene* in red, *ORF1Ab* in blue, and internal control in green.

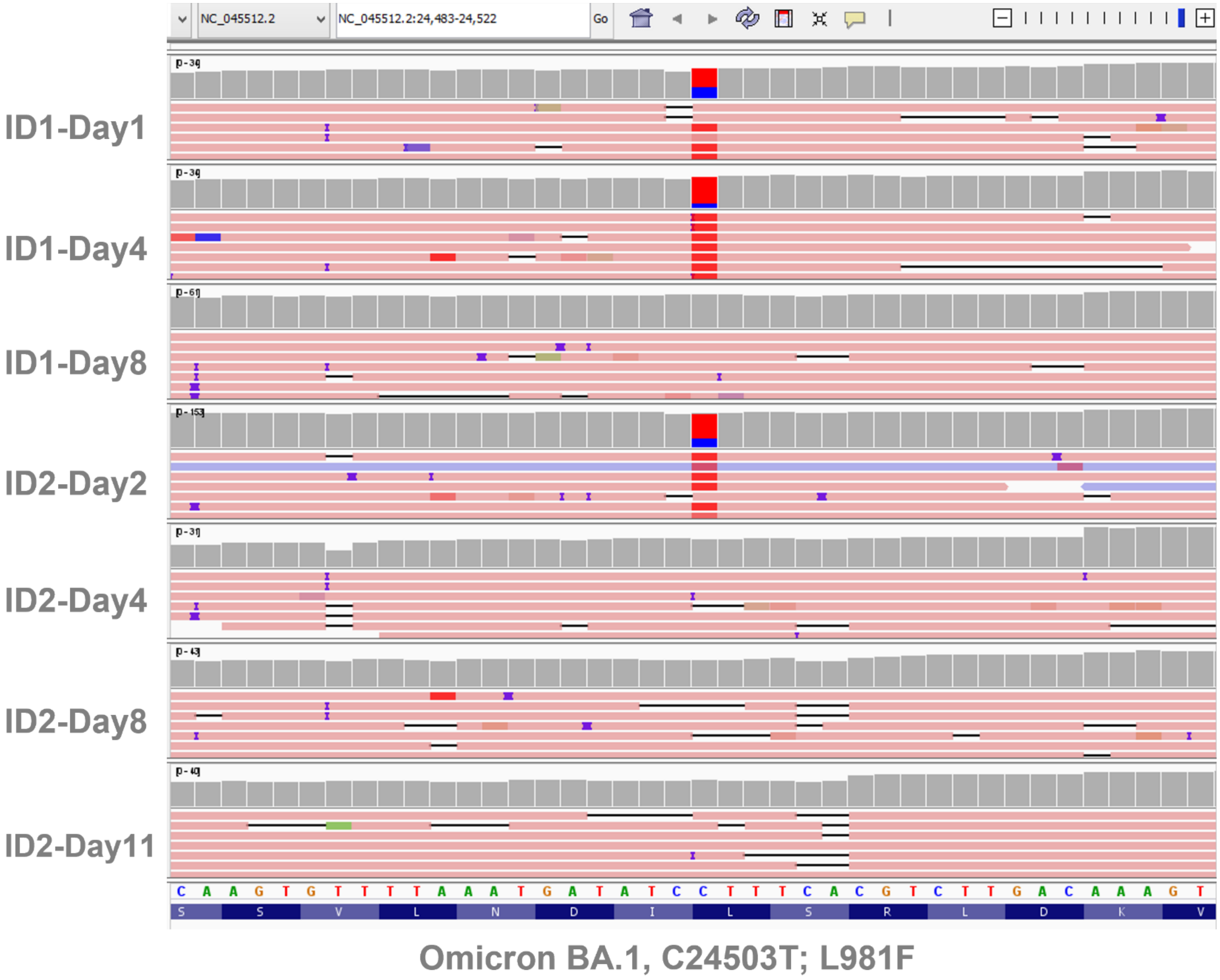

Supplementary Figure 2. Representative browser view of the C24503T (L981F) region specific for the Omicron BA.1 variant.

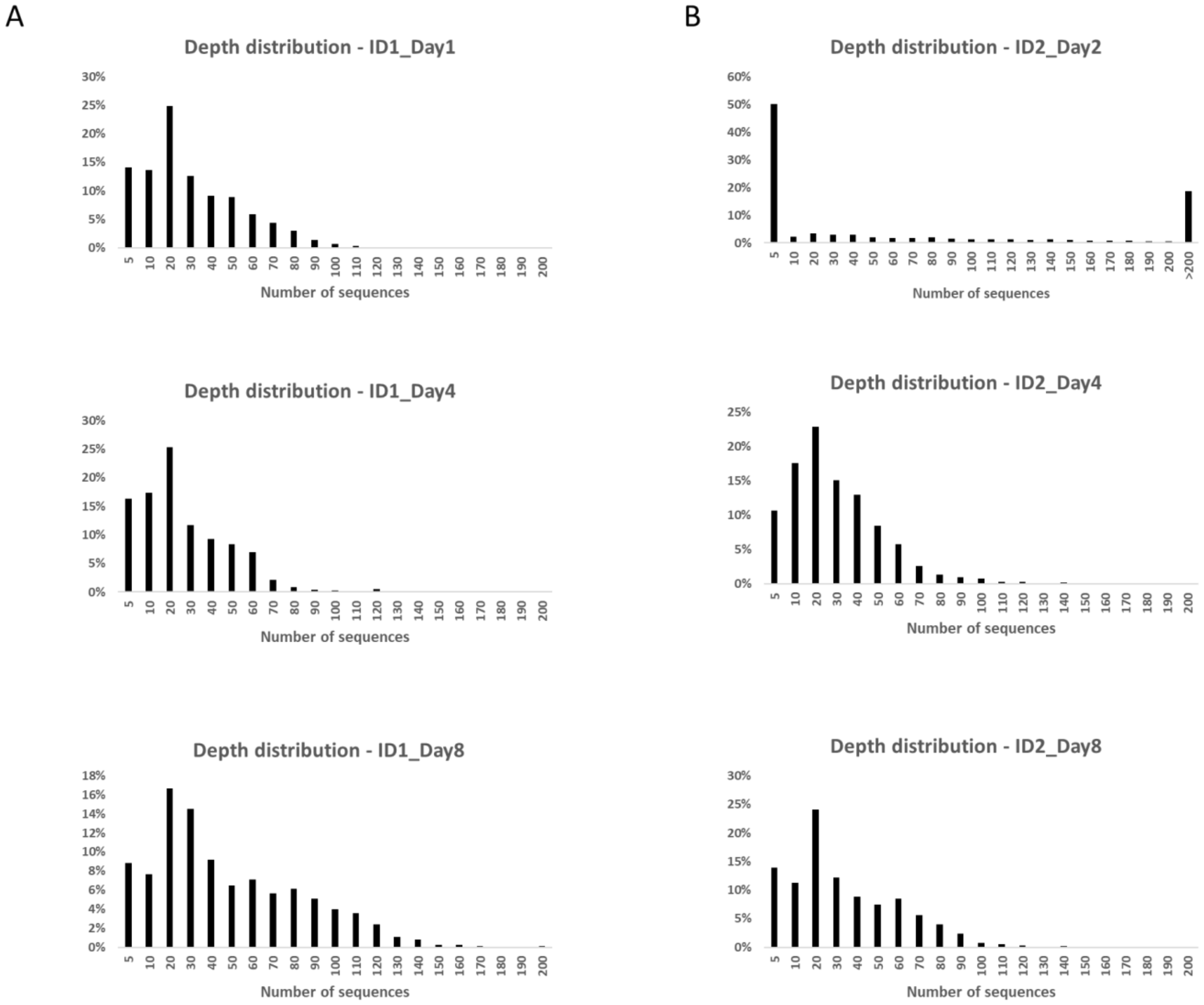

Supplementary Figure 3. Distribution of genome coverage of all base pairs in A) Individual 1, B) Individual 2. The average genome coverage of ID1_Day1 was 28x, ID1_Day4 was 23x, ID1_Day8 was 44x, ID2_Day2 was 121x, ID2_Day4 was 26x, ID2_Day8 was 35x.
